# Case Report: Dupilumab therapy for immune checkpoint inhibitor-induced bullous pemphigoid enables dual immunotherapy initiation in progressive malignant melanoma

**DOI:** 10.3389/fonc.2025.1613552

**Published:** 2025-09-25

**Authors:** Janine Grüninger, Saskia Lehr, Frank Meiss, David Rafei, Franziska Schauer

**Affiliations:** Department of Dermatology and Venereology, Medical Center - University of Freiburg, Faculty of Medicine, University of Freiburg, Freiburg, Germany

**Keywords:** malignant melanoma, eosinophilia, immune checkpoint inhibitor, NRAS, Th2 (type 2) immune responses

## Abstract

Immune checkpoint inhibitors (ICIs) targeting PD-1, PD-L1 and CTLA-4 have transformed the treatment of malignant melanoma, significantly improving patient survival rates. However, these therapies often result in immune-related adverse events, with cutaneous toxicities being the most prevalent. One such irAE is bullous pemphigoid (BP), which is rare but challenging, and is characterised by autoantibody-mediated blistering at the dermo-epidermal junction. ICI-induced bullous pemphigoid (irBP) affects around 0.6% of patients and presents a therapeutic challenge as it requires the management of both the autoimmune response and the underlying malignancy. Recent research has highlighted the role of Th2 cytokines, particularly interleukin-4 (IL-4) and IL-13, and eosinophils in the pathogenesis of BP and irBP. Dupilumab, a monoclonal antibody that targets the IL-4 receptor alpha subunit, inhibits IL-4 and IL-13 signalling. In this report, we present a case of irBP in a patient with metastatic melanoma who was successfully treated with Dupilumab. Following resolution of the autoimmune skin toxicity, the patient was re-challenged with dual ICI therapy (Nivolumab and Ipilimumab), which remains the recommended first-line treatment for metastatic melanoma. This case highlights the potential of Dupilumab as a steroid-sparing option in the management of irBP, enabling continued oncological treatment.

## 1 Introduction

The use of immune checkpoint inhibitors (ICI) has become standard of care for therapeutic approaches in the treatment of malignant melanoma (1). By targeting key regulatory proteins such as PD-1, PD-L1, and CTLA-4, these agents enhance anti-tumor immune responses and have significantly improved patient outcomes, leading to a marked reduction in overall mortality (2, 3). Despite these benefits, ICIs are associated with immune-related adverse events (irAEs), which affect up to 96% of treated patients (4). Among these, cutaneous side effects are the most common irAEs, occurring in 46-62% of cases (4). These skin-related toxicities commonly present with pruritic, eczematous, maculopapular, lichenoid, or bullous exanthema.

ICI-induced bullous pemphigoid (irBP) is an autoimmune-blistering disease triggered by autoantibodies targeting hemidesmosomal proteins located at the dermo-epidermal junction (5). The incidence of irBP is low, affecting approximately 0.6% of 5,636 patients treated for non-melanoma skin cancer (NMSC) or melanoma (6). The management of irBP is complex due to the underlying malignancy, as the need to control both the tumor and the autoimmune reaction represents a therapeutic challenge. Recent studies have revealed that Interleukin-4 (IL-4), Interleukin-13 (IL-13), and eosinophils play a significant role in the pathogenesis of bullous pemphigoid (7, 8). Dupilumab, an IL-4 receptor alpha antagonist, has demonstrated efficacy in reducing IL-4 and IL-13 signaling in various Th2-driven diseases and is now FDA-approved for the treatment of bullous pemphigoid (9). Therefore, it also represents a promising therapeutic option for irBP (10, 11). We here report on a patient with irBP successfully managed with Dupilumab, followed by re-exposure to dual immune checkpoint blockade (Nivolumab and Ipilimumab), which constitutes the current guideline-recommended first-line therapy for patients with unresectable or metastatic (stage IV) melanoma.

## 2 Case report

**Figure 1:**
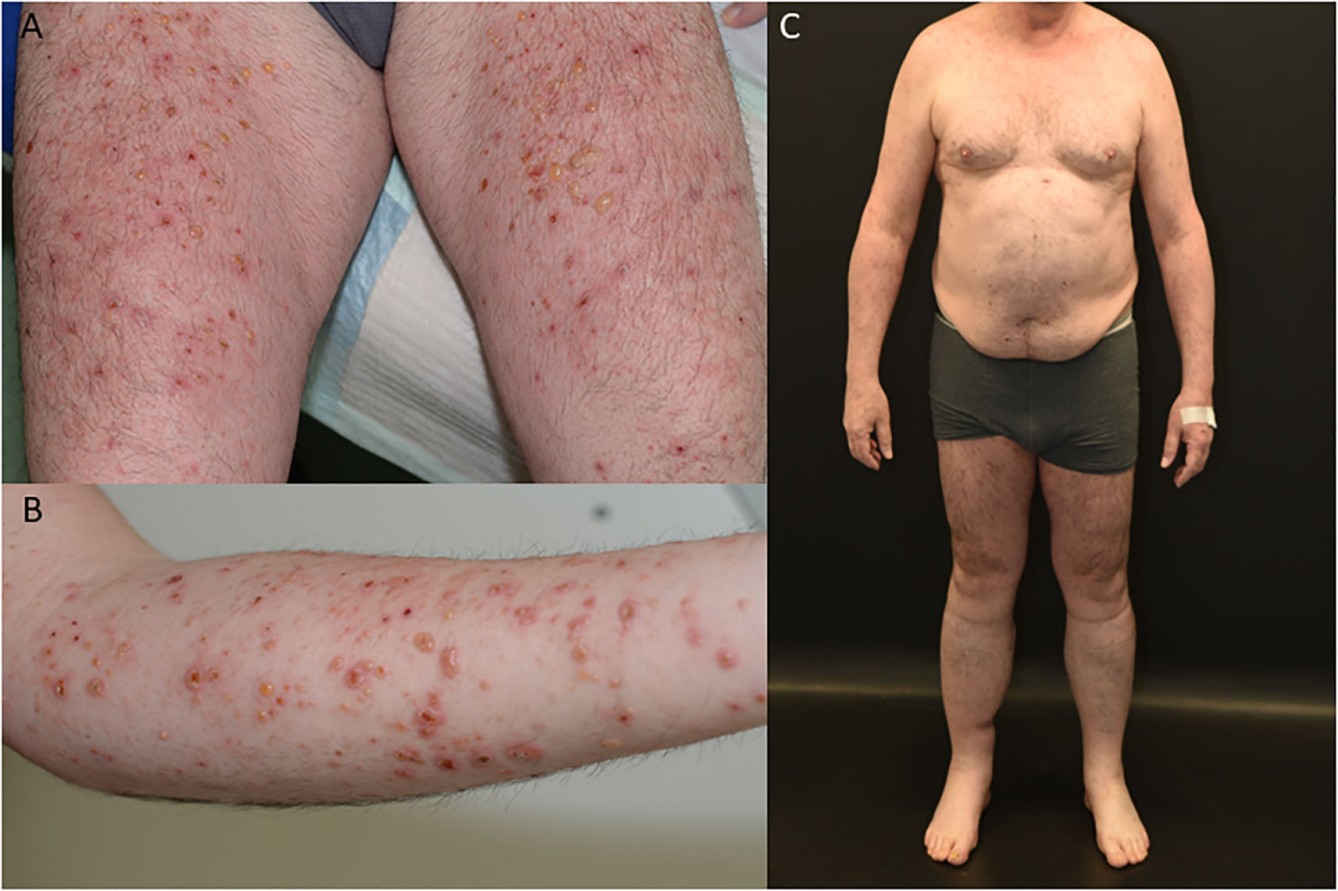
Clinical findings of patient with bullous pemphigoid **(A, B)**, patient in complete remission on Dupilumab therapy **(C)**.

**Figure 2:**
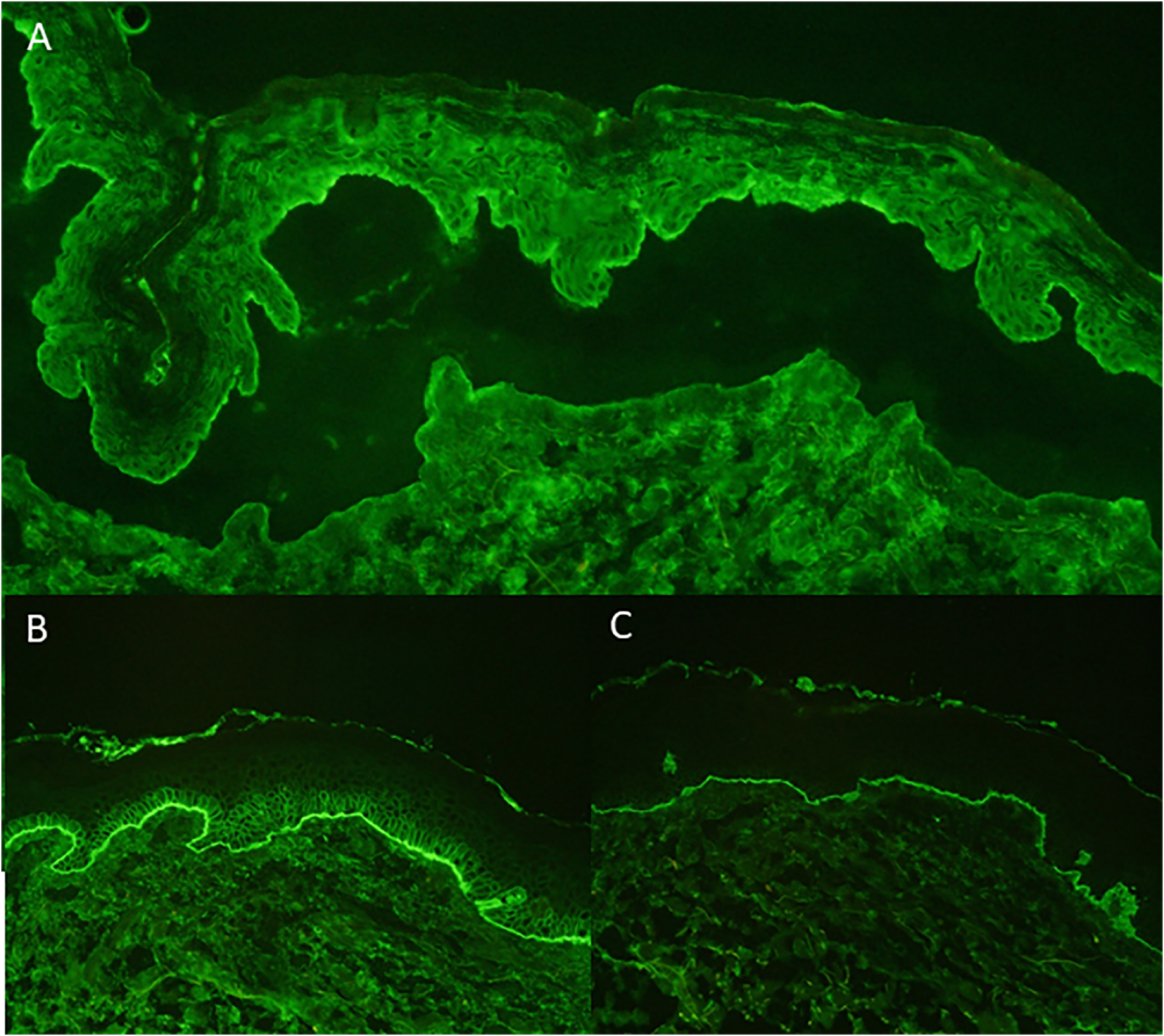
Indirect Immunofluorescence: linear IgG deposition in blister roof on salt-split-skin **(A)** Direct Immunofluorescence: linear deposition of IgG at basement membrane **(B)** Direct Immunofluorescence: linear deposition of C3 at basement membrane **(C)**.

A 59-year-old European man was treated for stage IIIC (AJCC 2017, 8. edition) malignant melanoma of the right calf (pT1b pN3c cM0) with Nivolumab 480mg every four weeks to reduce the risk of disease recurrence after complete resection. After seven months of successful treatment, the patient developed a bullous rash (CTCAE grade 3, v5.0) (**Figures 1A, B**). He had no prior documented history of autoimmune disease. Skin biopsy showed a subepidermal split with numerous eosinophils and a perivascular inflammatory infiltrate. Direct immunofluorescence microscopy revealed linear deposition of IgG and C3 at the basement membrane zone and the indirect immunofluorescence of split skin test showed IgG antibodies localized to the epidermal side of basement membrane zone (**Figures 2A–C**). ELISA testing confirmed the presence of autoantibodies against BP180 (63 Units/ml, cutoff <9 Units/ml, MBL^®^), while BP230 was negative.

**Figure 3:**
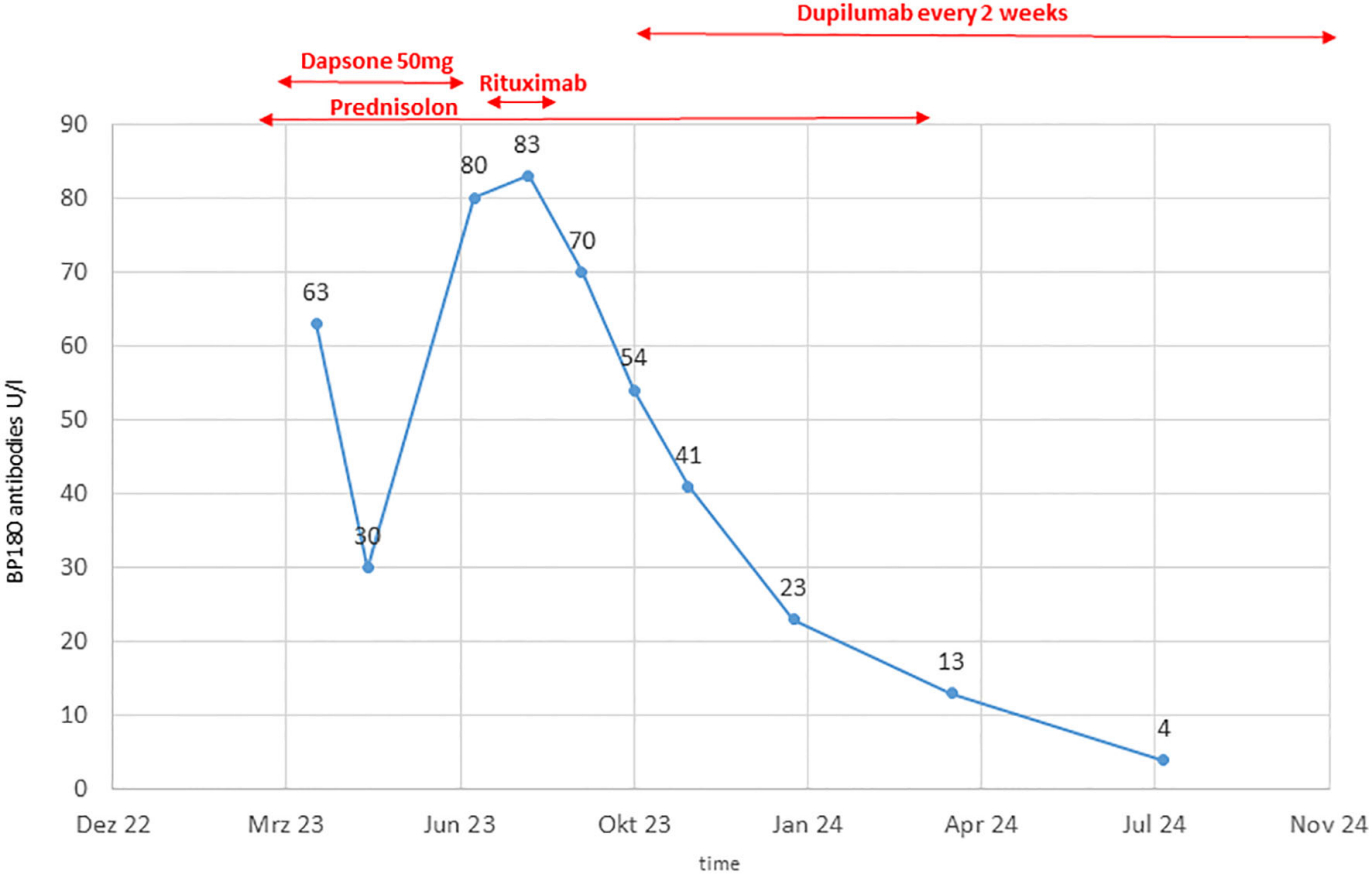
BP180 antibody level course over time.

The patient was initially treated with oral prednisolone 0.7 mg/kg BW, which was tapered due to a positive response over 15 weeks. Dapson 50 mg was added but showed limited efficacy, necessitating a switch to Rituximab after 20 weeks. After two doses of Rituximab (1000 mg each), disease control was not achieved, as indicated by increasing BP180 antibody levels up to 83 Units/ml and ongoing clinical disease activity (**Figure 3**).

**Figure 4:**
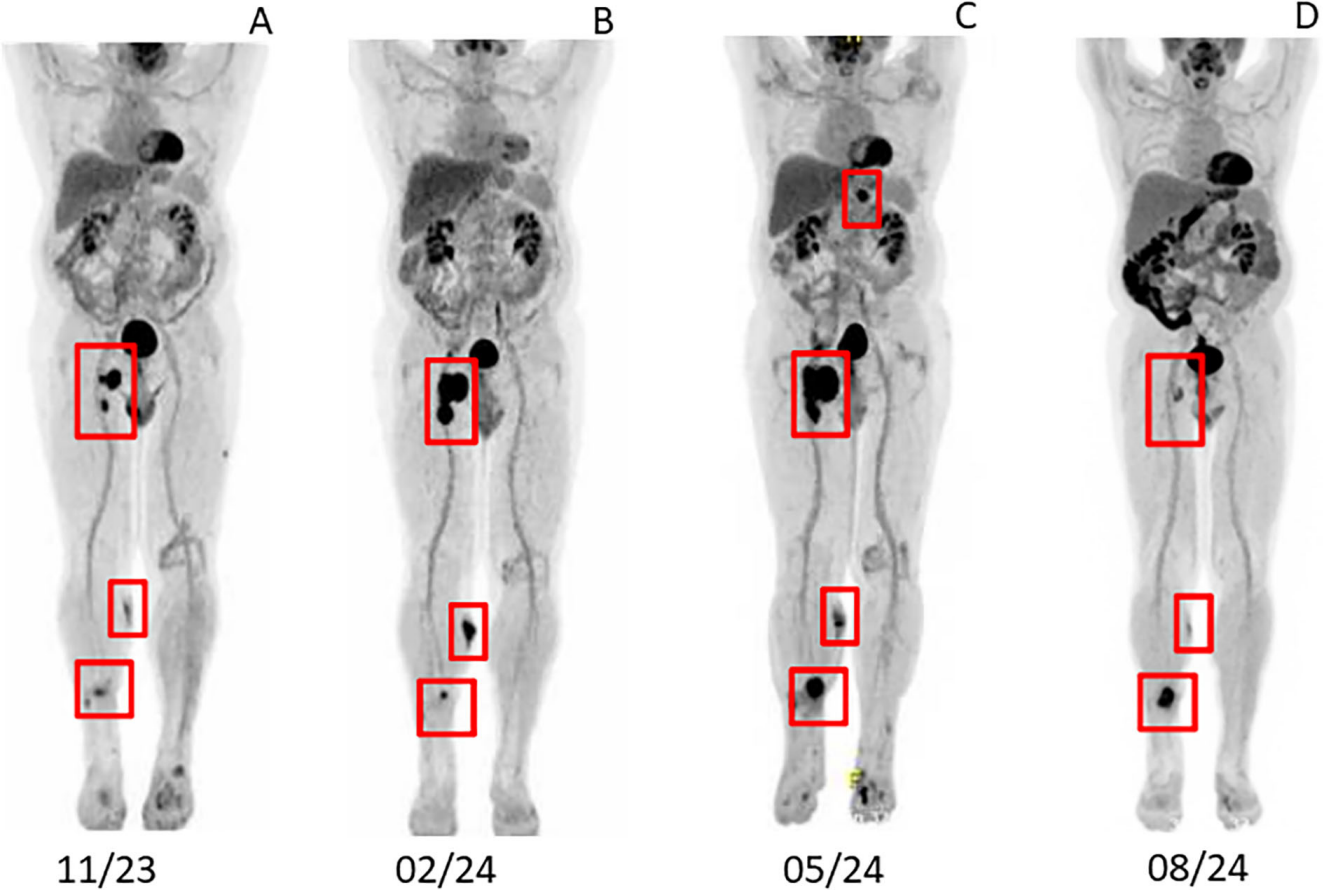
Staging examination via PET-CT **(A)** after adjuvant therapy with Nivolumab 11/2023 **(B)** after treatment with Darcabazine and intralesional Interleukin-2 02/2024 **(C)** after Ribociclib in combination with Binimetinib 05/2024 **(D)** after two doses of Nivolumab in combination with Ipilimumab 08/2024.

Due to the failure of established therapies, subcutaneous Dupilumab 300 mg every two weeks, was initiated as an individual treatment approach. After 5 weeks, the patient’s BP180 antibody level dropped to 54 Units/ml, and no new skin lesions developed (**Figure 1C**). Under continued Dupilumab therapy for 11 months, antibody levels gradually decreased to 4 Units/ml (**Figure 3**). Due to immunotherapy associated side effects and new cutaneous and lymphatic metastasis (**Figure 4A**), the oncological treatment had to be switched to Dacarbazine 1000mg/m^2^ BSA every 3 weeks. For cutaneous metastases at the right calf we additionally added weekly intralesional local injection of Interleukin-2 (IL-2) with a dose of 2.0ml (6 MIU/ml) over a period of 10 weeks. Due to further tumor progression under Dacarbazine and IL-2 treatment (**Figure 4B**), the therapy was switched to a combination of Binimetinib (MEK inhibitor) and Ribociclib (CDK4/6-Inhibitor) based on the NRAS exon 3 mutation (p.Q61R) (12). Nevertheless staging examinations (**Figure 4C**) after 6 months revealed progressive disease with increasing cutaneous and lymph node metastases, along with the emergence of new pulmonary and osseous metastatic lesions. This created significant oncologic therapeutic pressure, necessitating an escalation of therapy with the combination of Nivolumab and Ipilimumab (**Supplementary Figure 1**), while the patient was still receiving Dupilumab. After two doses of dual immunotherapy, the patient did not develop any new blisters related of irBP, however autoimmune colitis (ir-colitis, CTCAE grade 2, v5.0) and an autoimmune hepatitis (ir-hepatitis, CTCAE grade 1, v5.0) was observed. Follow-up staging examinations demonstrated a very good response of the melanoma metastases (**Figure 4D**). Due to the steroid-refractory course of the ir-colitis, immunotherapy had to be discontinued, and the patient received an extracorporeal photopheresis (ECP) as part of an ongoing study at our cancer center. ECP treatment was administered 5 times every 2 weeks and resulted in complete remission of ir-colitis (13). Although the immunotherapy could only be administered with two doses of dual immunotherapy a stable oncologic disease has been observed since 9 months now.

## 3 Discussion

Cutaneous side effects are the most common immune-related adverse events (irAEs) caused by immune checkpoint inhibitors in up to 96% of patients (4). Besides different types of exanthema, rare skin toxicity includes bullous dermatoses in 0.3-0.6% of cases (6, 14). Diagnostic accuracy of autoimmune bullous diseases is enhanced by current guidelines, which define the diagnosis of BP based on the detection of both tissue-bound and circulating autoantibodies, assessed through direct and indirect immunofluorescence as well as ELISA testing (15). Depending on the stage of the disease, not all diagnostic criteria may be met at initial presentation, which has been especially described in irBP (16–18). Early identification or exclusion of BP in patients presenting with ICI-induced exanthemas is of particular importance. In this context, timely skin biopsies and serial serological testing are essential. The timing of disease onset in relation to immunotherapy, the younger age of affected patients, and the clinical presentation - characterized by more extensive skin and mucosal involvement and initially presenting as a lichenoid exanthema -differs from that of idiopathic BP (18). While irBP can resemble idiopathic BP, recent data indicate distinct differences, including a lower frequency of elevated BP230 autoantibodies in irBP (18).

A plausible pathogenetic mechanism for the development of irBP involves autoreactive T cells targeting BP180, a key transmembrane component of the dermoepidermal junction within the skin and co-localized in the tumor (5). The early and elevated detection of BP180 autoantibodies in irBP likely reflects the enhanced T cell activation triggered by immune checkpoint inhibition. While BP180 is directly accessible to the immune system, BP230 remains intracellular and becomes antigenic primarily upon cell damage (11). Studies also show that B cells, which express PD-1 and PD-L1 may be directly activated by anti-PD-1 therapy independently of T-cell stimulation (19). Moreover, these pathogenic B cells could be indirectly activated by T-follicular regulatory cells, which are dysregulated by anti-PD-1 therapy (20). The efficacy of Rituximab, a B cell depleting agent, supports the hypothesis of B cell involvement (6). Additionally, eosinophils play a significant role in blister formation through anti-BP180 IgE (7), with IL-4 crucial for their recruitment and feedback loop promoting IL-4 production (21). The presence of IL-4 and IL-13 in skin lesions (22), along with a higher frequency of skin-homing cutaneous lymphocyte-associated antigen-positive IL-4- and IL-13-producing T cells in skin blisters (8), provides additional support for the hypothesis that IL-4 and IL-13 are essential for the pathogenesis of BP (11).

IrBP poses therapeutic challenges due to balance effective tumor control with the management of irAEs. Current guidelines for managing irBP lack specific treatment recommendations (15). It often requires discontinuation of ICI, which is not desirable, and the initiation of prolonged immunosuppressive therapy (23). Systemic glucocorticosteroids are the first-line treatment for immune-related bullous pemphigoid (irBP); however, some cases are associated with long-term use or are glucocorticosteroid-resistant, which may interfere with tumor response.

Dupilumab blocks the shared receptor component for IL-4 and IL-13, key and central drivers of type 2 inflammation. It was recently approved by the FDA for idiopathic BP (9, 24). Several reports of patients with irBP successfully treated with Dupilumab have been published (10, 25). Thus Dupilumab, may represent an effective treatment option in irBP with minimal side effects and should be considered if immunosuppressive therapy should be avoided. In our case the efficacy of Dupilumab even allowed reexposure to dual immunotherapy without exacerbation of irBP.

Our patient demonstrated tissue eosinophilia but no peripheral blood eosinophilia (PBE) during the development of irBP. However, both tissue and peripheral eosinophilia can occur in irBP, either in association with the tumor microenvironment or as a result of oncologic treatment (26). Several mediators, such as IFN-γ, IL-5, IL-33, and CCL11, can enhance eosinophil-mediated cytotoxicity, consistent with the role of eosinophil-derived factors in eosinophil-driven tumor cytotoxicity (26). In melanoma PBE has been described as a biomarker for prognosis of melanoma patients and correlates with the response to immunotherapy (27) To date, eosinophil-depleting therapies using monoclonal antibodies targeting IL-5 and IL-4/13 have not shown evidence of an increased prevalence of neoplasia (25, 28). However, long-term effects remain unclear, and prospective irAE registries are needed to better assess this risk.

Besides elevated IL-4 and IL-13 levels, recent studies comparing gene expression between irBP and BP have revealed increased expression of PD-1, CTLA-4, and LAG-3 in patients with irBP (18). Elevated lymphocyte activation gene-3 (LAG-3) has been linked to poor overall survival in various tumors, including uveal melanoma (29). Since combined anti-PD-1 and anti-LAG-3 inhibition is already approved for metastatic melanoma treatment, the heightened LAG-3 gene expression in irBP could offer another treatment avenue for irBP.

## 4 Conclusion

The treatment of irBP remains a therapeutic challenge, particularly in the presence of an underlying and progressive malignancy. Dupilumab, by inhibiting Th2 cytokines IL-4 and IL-13, represents a promising therapeutic approach. This case demonstrates the potential for using Dupilumab to manage irAE and progressive tumor disease by allowing reexposure to immunotherapy. Further studies are needed to determine the optimal duration of Dupilumab therapy in irBP patients and to evaluate long-term safety and efficacy.

## Data Availability

The datasets presented in this article are not readily available because no datasets used. Requests to access the datasets should be directed to franziska.schauer@uniklinik-freiburg.de.

## Supplementary material

Supplementary Figure 1 Therapeutic schedule of the patient with progressive malignant melanoma and irAEs.
