# Supplementary figures and images for: Case Report: Dupilumab therapy for immune checkpoint inhibitor-induced bullous pemphigoid enables dual immunotherapy initiation in progressive malignant melanoma
Source: Front Oncol. 2025 Sep 25;15:1613552. doi: 10.3389/fonc.2025.1613552 (PMC12507624; doi:10.3389/fonc.2025.1613552)

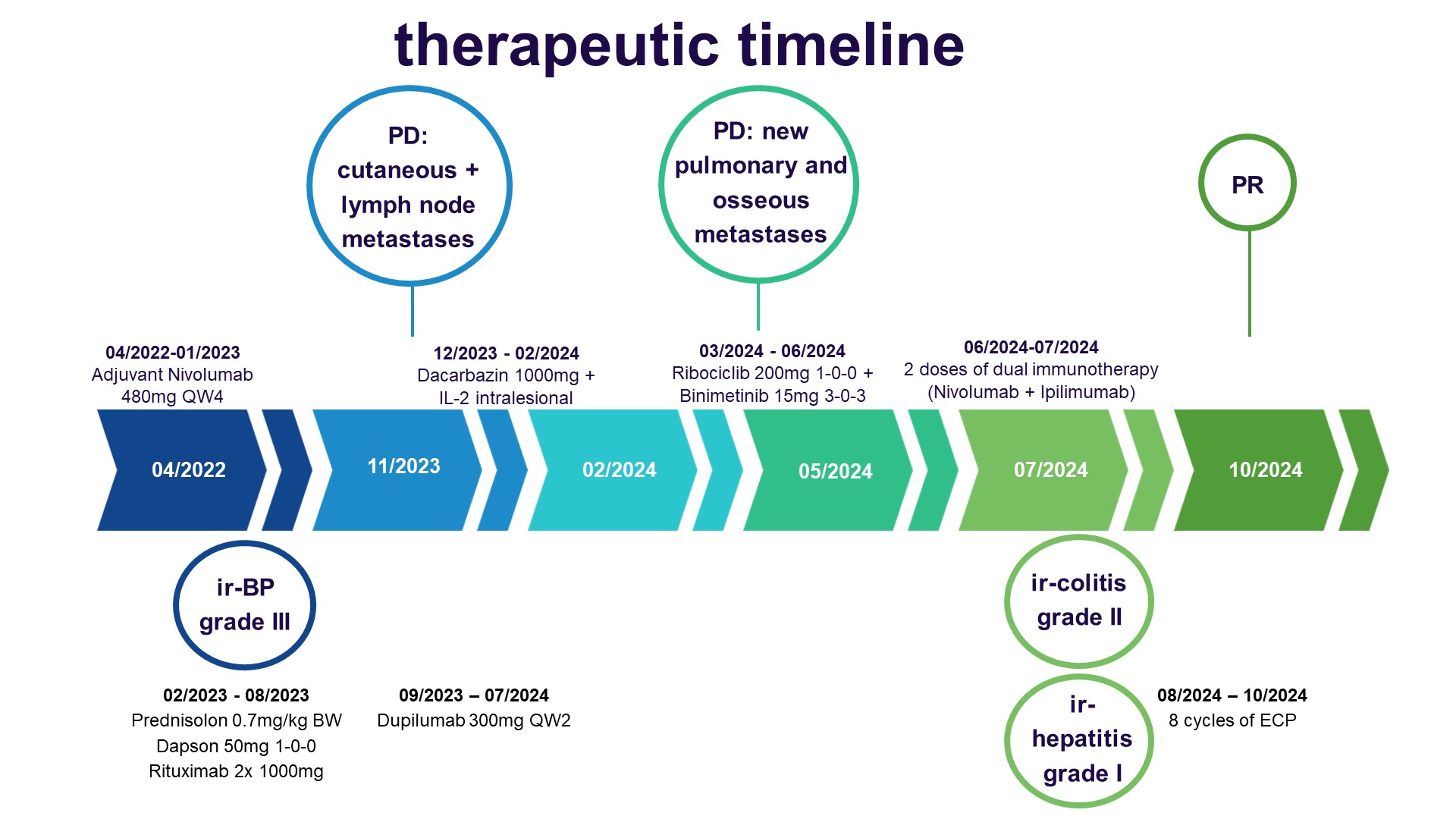

Supplement: Supplementary Figure 1 — Therapeutic schedule of the patient with progressive malignant melanoma and irAEs. [file Image1.jpeg]
